# The Foreign Journals: Surgery

**Published:** 1840-07

**Authors:** 


### SURGERY.

### *Calculus, weighing* 23½ *ounces, formed in the Urethra.* By Dr. Da Luz.

J. L. æt. thirty, a fisherman near Lisbon, had had a difficulty of passing urine from his infancy. From the same period also he had had a hard tumour in the perineum; and at a later date a fistula, which had healed spontaneously. The tumour, however, continued to increase, though without producing any inconvenience except from the distension of the parts around it, which becoming at last excessive, the patient came to the Hospital of San Joseph, at Lisbon.

At this time (1836) the tumour which projected into the perineum had a diameter of 5½ inches; its form was nearly that of a large pear; it occupied the whole scrotum, and caused great distension of its integuments. The testicles lay one on each side of the tumour; the penis in a normal state was in its front. The perineum, which was hard and very prominent, presented several irregular cicatrices, and here and there excoriations from the contact of a sedimentous, purulent, and fætid urine which constantly flowed by drops from the meatus. The catheter easily detected a foreign body in the urethra.

Two deep semi-elliptical incisions being made in front of and below the tumour, the base of a calculus was exposed on which there was a furrow corresponding to the septum scroti, which gave it a bilobed form. The incision being then prolonged towards the perineum the stone was drawn out with forceps, leaving a great cavity, at the bottom of which the prostatic portion of the urethra and the neck of the bladder were seen so dilated that the operator was able to introduce his finger into the bladder. The patient left the hospital in twenty-nine days; the operation was followed by no bad consequences, but a fistula remained in the perineum which healed in the course of the following year.

The calculus, which there is every reason to believe had passed when small from the bladder into the urethra where it became fixed, was, as already said, pear-shaped; the smallest portion of it corresponded to the neck of the bladder, the larger end was enveloped by the membranes of the scrotum; two depressions were observed in its sides, where the testicles had rested; and two grooves in the under surface, one of which fitted upon the septum scroti, while the other extended along the whole length of the stone, and permitted the flowing of the urine. The composition of the calculus was phosphate of lime; it measured 5 inches in its longest diameter, and 3½ inches transversely; its weight was 23½ ounces.

*Revue Médicale. Février,* 1840.
(*From the Lisbon Journal of the Medical Sciences. Feb.* 1839.)

---

*Extirpation of nearly the whole of the Clavicle.* By Prof. REGNOLI, of Pisa.

THE subject of this operation was a carrier, thirty-four years old, who had always enjoyed good general health. In August, 1838, while lifting a sack of grain, he felt a pain in his left shoulder which ceased after a short time. Not long after, however, it reappeared in the region of the clavicle, affecting him especially every time that he returned to his work; and at the end of about ten days it became incessant and prevented his sleeping. The suffering part, as well as those adjacent to it, now rapidly swelled, and the application of leeches and other means were ineffectual in restraining the inflammation that ensued; suppuration took place, and the matter made its way out by ulceration through the skin. In November, when the patient presented himself at the Clinic at Pisa, a considerable extent of the clavicle was found to be in a state of necrosis; the patient was emaciated and had hectic fever; and after incisions over the dead bone and some other means had been employed without any benefit, it was determined to extirpate the clavicle.

For this purpose, the patient being seated, an incision was made through the track of the several ulcers over the clavicle and prolonged to each extremity of that bone, so as to pass a few lines over each and especially over the sternal end. The bone being thus exposed a portion of its diaphysis was found almost isolated, and this being seized with strong forceps was with little difficulty extracted. The two extremities still remained; the sternal was in a state of necrosis, but the acromial appeared healthy: the former was disarticulated by cutting with scissors, and the latter (which was but a small portion) was left. The operation was rendered peculiarly difficult by the tissues having lost all their natural appearance so that it was impossible to recognize any anatomical relations for the guidance of the knife. There was no bleeding from any considerable vessel nor was any artery tied; the subsequent progress of the case presented nothing remarkable, except that in the inflammation which followed the operation the acromial portion of the bone became necrosed, and some pieces of it were extracted. The extirpated portion of bone was ultimately replaced by a dense tissue of fibrous consistence: the patient was enabled to move his arm in all directions without any difficulty, and, with the exception of slight weakness of the limb, the consequence of its want of exercise, everything was restored to its normal condition.

*Annali Medico-Chirurgici di Roma.* Vol. i. 1839.

---

*On Subcutaneous Wounds.* By M. JULES GUERIN.

IN these papers, which form a summary of the very bold practice of the author in this novel branch of surgery, his chief endeavour appears to be to point out an explanation of the peculiar pathology of subcutaneous wounds; that is, of wounds of considerable extent made in all the tissues beneath the skin through a very narrow opening in it. The following abstract, however, contains what appears to us a more useful part of the memoir, and by showing to what an extent the practice may be safely carried will prove that it should be adopted in every possible case in which inconvenience is feared from exposure of the wounded tissues to the air.

"I took (says M. Jules Guerin) two dogs of moderate size, the one young the other an adult. In the former I made a subcutaneous division of the mass of muscles in the posterior grooves of the vertebral column in three places; one in the superior scapular region comprising the trapezius, rhomboideus, serratus posticus, sacro-lumbalis, and longissimus dorsi, and the two layers of inter-transversales; a second through all the muscles and aponeuroses in the middle of the back; and a third at the level of the third lumbar vertebra, implicating in like manner the whole width of the sacro-lumbalis and longissimus dorsi. After these, I cut transversely beneath the skin through the flesh of the posterior and upper part of each thigh quite down to the bone, so as to divide the glutæus maximus, biceps, semitendinosus, semimembranosus, vastus internus, adductor magnus, and the sciatic nerve and corresponding vessels. Each of these sections was made with a very narrow knife introduced through a small hole in the skin at some distance from the main incision; and to secure more effectually the exclusion of the air I closed the openings in the skin with sutures.

"In the adult dog I made a long wound under the skin, extending from the lower part of the neck to the sacrum, parallel to the line of spinous processes, so as to divide all the muscles down to the arches of the vertebræ. I then made a transverse section of all the tissues at the back of the left thigh from beneath the skin to the bone, as in the former case, and closed both the little openings in the skin with sutures.

"In the first dog immediately after the operation there were slight effusions of blood under the skin at the divisions of the vertebral muscles; and a considerable quantity of fluid poured out at those of the muscles and vessels of the thighs. In the second dog there was an effusion without any considerable swelling all along the hollow of the vertebræ corresponding to the wound, and an immense effusion in the thigh, so that the size of the buttock was doubled, and the skin was made round and tense.

"Both dogs passed the night quietly, and on the next day neither of them had the least appearance of fever. On the day after they appeared better, eating and drinking as usual, and beginning to move about freely. No trace of the operations on the muscles of the spine could be found in either of them, and in the thighs there was much less tumefaction and no pain on pressure. On the fourth day the oldest of the dogs seemed to have forgotten his mutilations; he was walking and running and jumping about all day, exactly as he used to do before the operations. Next day, however, his wounds were a little tender; but on the following he was again perfectly well and every tissue that had been divided was united and perfectly restored to its former functions.

"In the younger dog the phenomena were still more interesting; none of the five wounds excited the least sign of inflammatory reaction: on the fourth day he began to raise himself up on his hind legs (which had of course been completely paralyzed), and on the eighth he could walk; he now presents scarcely the least trace of paralysis.

"Here, therefore, are examples of subcutaneous wounds implicating a great extent of tissues, dividing muscles, aponeuroses, vessels, and nerves, and followed by considerable effusions beneath the skin, and yet cured, nay even organized immediately almost, as if there had been but a simple lesion of the skin itself."

On the assurance afforded by the results of these experiments M. Guerin has divided the human muscles to an almost equal extent in thirty persons affected with curvature of the spine. The operation was followed in all, he assures us, with considerable benefit; and on only one occasion did there result from it the least inconvenience; in that case there was an unimportant diffusion of air along the track of the wound.

*Gazette Médicale. Avril* 1, 1840.

*Remarkable Case of Hernia of the Fallopian Tube, with Dropsy of the Hernial Sac.*
By M. A. BERARD.

MADAME B. æt. forty-five, has habitually enjoyed good health, complaining only within the last few years of occasional weakness in the loins and transient pain in the lower part of the abdomen; has had two natural accouchements and has never worked at any laborious trade. Two years since she perceived a small tumour, disappearing on pressure, in the right groin, for which she was recommended by her medical attendant to wear a bandage; this advice she neglected; the tumour slowly increased in size, continuing to be reducible. In December, 1837, its growth became more rapid and the abdominal pain more acute than usual. At this period, M. Bérard and another practitioner ascertained the following particulars: in the right groin is seated a tumour larger than a hen's egg, stretching somewhat towards the abdomen and right labium, with a broad base and even surface, except internally and superiorly, where a mammillary body about the size of the top of the finger protrudes above the rest; here, too, the skin is adherent, exceedingly attenuated, and slightly bluish; elsewhere the integuments of the swelling preserve their natural characters. The tumour is painless, irreducible, and unchanged in size by long-continued pressure in various directions; percussion shows that it contains no gas; it is manifestly fluctuating in every part, and, judging from its perfect transparency, is filled with serosity. The patient affirms that the tumour still returns into the belly, when she has lain for some time in bed. A hard round body, of the size of a turkey's egg, protrudes above the pubis; by a vaginal examination this is found to be developed in the body of the uterus. From these facts we inferred that the patient had one or more fibrous tumours in that organ, and that on these depended the abdominal pain and weight in the loins and pelvis. We set aside all idea of strangulated hernia, and considered the inguinal tumour caused by a serous cyst developed in the part, or by an old hernial sac, closed by adhesion at the neck and affected with dropsy. The patient becoming anxious for its removal, M. Marjolin's opinion was had; and the diagnosis now made was: femoral hernia of long standing; dropsy of the sac, which contains, besides, some abdominal viscus, probably a piece of omentum; complete adhesion of the latter with the neck of the sac, whereby the passage of the contained liquid into the abdomen is prevented. The tumour was now punctured with a trocar at its most prominent point, and from six to eight ounces of citron coloured, frothy serosity, becoming gelatinous by heat, drawn off. After this evacuation a round body, of the size of a small nut and irreducible, was distinctly felt in the femoral ring, and ceased to be felt behind the crural arch. Compresses steeped in aromatic wine were spread on the sac, to excite adhesion of its sides. In the afternoon a fit of shivering came on and the tumour grew painful; the following morning the latter had refilled; skin red and hot; general prostration. (twenty-five leeches to the tumour.) *Third day.* Abdomen painful and distended; vomiting. The tumour, incised freely, discharges much lactescent serosity. *Fourth day.* Erysipelas about the tumour, (thirty leeches.) Pallor, pulse small in the day, rose in the night. *Fifth day.* The sac suppurates. *Sixth day.* Symptoms of peritonitis evident, (mercurial friction, blisters to the thighs.) *Seventh day.* Death. *Dissection.* Sero-purulent effusion in abdomen, false membrane on the intestines, &c. The interior of the cavity of the tumour is lined with an albuminous exudation, and communicates by a perfectly free opening with the peritonæal cavity, behind Poupart's ligament; this is evidently the neck of a hernial sac: the opening is two or three lines wide and corresponds to the femoral ring. *The hernial sac contains nothing but the Fallopian tube in a state of considerable hypertrophy;* there is no adhesion between the tube and the interior of the sac, but the former is closely united to the anterior part of the circumference of the latter; in the space where these parts are not adherent there exists a free communication between the peritoneum and the interior of the cavity containing the serosity. The ovary of the right side occupies its usual position in the pelvis; the tissue of the uterus, otherwise healthy, is distended by an enormous fibrous tumour. From the appearance of the parts it is very possible that he liquid in the sac may have gradually oozed during the night through the opening; though the sudden pressure used in the taxis, by forcing the displaced tube into the narrow passage, may have stopped the latter up completely. In this manner the assertion of the patient respecting the disappearance of the tumour in the night, is reconcilable with the fact that it was irreducible by art.

[Cases of hernia of the Fallopian tube without the uterus or ovary are extremely rare; the erudite editor of the journal from which we make this extract has only been able to discover two such cases, and the nature of one of these cases is not wholly incontestable.]

*L'Expérience.* No. xcii. *Avril,* 1839.

---

*Reduction of Strangulated Inguinal Hernia by the method of Dr. Hesselbach.* By Dr. Lyncker.

[The following case is well worthy of consideration: it belongs to the rude, rough, common-sense surgery of the old time, now, perhaps, too indiscriminately banished from practice.]

Dr. Lyncker attended (April 25) a cachectic old woman, who had suffered for four days from a strangulated inguinal hernia. The belly was swollen, but not painful; the seat of the rupture was painful, but without any other perceptible change. The taxis was ineffectually employed. Leeches, cold applications, and tobacco clysters were made use of. On the following day the taxis was again resorted to but without effect.

During the night of the 26th and on the 27th of April, there came on fæcal vomiting, with great restlessness, anxiety, and debility; but there was no change in the situation of the hernia. The taxis was again carefully employed, but made no impression on the swelling. The patient would not submit to an operation. Dr. Lyncker bethought himself of the method of reducing intestinal ruptures, thus described by Dr. Hesselbach. "A strong man should place himself at the end of the bed on which the patient is lying, and, placing the legs of the patient over his shoulders, so that each knee of the patient shall rest upon one of his shoulders, his feet hanging downwards, shall then raise up the patient. The thighs of the patient are thus drawn upwards, his head and body resting upon the bed. In this position the taxis is to be repeated."

By placing the patient in the position above described, and reemploying the taxis, the ruptured bowel was suddenly reduced.

*Wochenschrift für die Gesammte Heilkunde.* No. xxvi. 1839.

---

*On Amputation of the Penis.* By M. Barthelemy.

"I published in 1829 the description of a new mode of proceeding in cases of amputation of the penis, the peculiarity of which consists in the introduction, before performing the section, of an elastic gum catheter, which is made to abut against the posterior wall of the bladder. Objections have been made to this plan: it has been said that the end of the catheter left in the bladder, after the amputation of the organ, must slip into that viscus; experience shows, on the contrary, that it springs forward from the elastic reaction of the bladder. The plan has also been declared useless; but experience destroys this objection also, for after the division of the penis, the urethra sinks inwards in such a manner that it is always difficult and has been *sometimes impossible* to find its orifice. The following facts prove this: M. Béclard, of Strasburg, amputated a penis in presence of M. Casimir Broussais, and being utterly unable to find the orifice of the urethra, was obliged on the following day to puncture the bladder through the rectum; the canula having accidentally escaped, it became necessary to puncture above the pubis, an operation subsequently several times repeated; the bladder was in a deplorable state, when the patient fell a victim to a most seasonable attack of smallpox. M. Gimelle having amputated a penis, was unable to find the urethra, and his patient died in consequence of infiltration of urine. MM. Mirmont and Bury were, in two cases, (and I have read of a similar one in the Lancet,) unable to find the canal for a quarter of an hour. Whether this difficulty depend upon retraction of the mucous membrane of the urethra, like that observed to take place in the arteries of lacerated parts, or upon investment of the orifice of the canal by the neighbouring spongy tissues, is not easy to determine; the important point is that such difficulty arises, and that it may be obviated by the precaution I recommend. In the performance of the operation it must be borne in mind that a pliable catheter is requisite, and that this must be carefully made to abut against the posterior wall of the bladder. An assistant should be placed to the right of the patient, and placing his left hand on the pubis, press the tissues by means of the index finger and thumb with some force against the catheter, while with his right he supports the part of the instrument protruding from the urethra. If the disease have spread too close to the pubis to admit of the fingers being placed as just directed, the canal may be pressed from below upwards behind the scrotum. The operator then cuts with a small amputating knife through the penis and catheter at a single stroke. The assistant then ceases to press upon the latter, which immediately springs forwards; the operator then draws it out about three inches and fixes it in its place in the ordinary way." Five cases are referred to wherein this plan was pursued with success.

*Gazette Médicale de Paris. Nov.* 1839.

---

*On the Facial Hemiplegia of Newly-born Children.* By H. LANDOUZY, M.D.

THIS affection is attributed by Dr. Landouzy to pressure exercised by the forceps upon the seventh pair of nerves. He then speaks of the conditions in the infant which he supposes capable of rendering this pressure efficient in the production of paralysis. In the adult, the projection of the mastoid process, of the external auditory canal, and of the sterno-mastoid muscle prevent any pressure upon the seventh pair at its exit from the pars petrosa; but in the newly-born child, the almost entire absence of the mastoid process, and the exceedingly small development of the external auditory canal permit the compression of the nerve by the forceps, especially when the parietal bone is the presenting part. But it is rarely that the pressure takes place in this direction. And it may be exercised upon branches instead of upon the trunk of the nerve; under which circumstances the symptoms will be found to vary in accordance with those portions of the nerve which have been compressed. Although Dr. Landouzy knows no other cause than this compression which produces the paralysis which he describes, he does not assert that other causes may not produce it; such, for instance, as tumours in the pelvis, irregularities in the pelvic bones, &c. And a compression by the forceps may have been sufficient to produce paralysis, without having left any marks on the face of the child, as is shown by a case here referred to. In connexion with this fact, Dr. Landouzy properly remarks, that "when we consider that the most distinguished practitioners readily admit the existence of effusions where the disease is a purely local affection of a nerve, the importance of insisting on this peculiarity in the facial paralysis of newly-born children becomes manifest, i. e. that the forceps may have exercised sufficient pressure upon the seventh nerve to paralyze it, without leaving any external evidence of its action." It is evident that a proper knowledge of the nature and origin of this disease is important, in order that no such remedies may be employed for its cure as would be injurious to the health of the infant, and which at the same time would have no effect in cutting short the disease. The difference in symptoms between this affection in the newly-born child and the adult is, that whilst in a state of rest, and during moments of perfect calmness, the expression of the child's face is scarcely at all changed. It is immediately after birth, and whilst the child is crying, that the first symptoms are noticed, and then they are particularly marked. The commissure of the lips is much deformed; the ala nasi is less dilated and less moveable than on the healthy side, the eyelids widely opened, whilst those of the sound eye are shut. All these appearances are increased with the increasing cries of the child. These marked appearances cease almost completely when the cries cease. But in the adult, the deformity although less during a state of repose is then also very decided. Another difference between the disease as happening in the child and in the adult is, that in the former, the symptoms spontaneously cease in a period varying from a few hours to two months. And it should be remembered that the treatment required, in the majority of cases, is simply hygiènic. These remarks on symptoms, progress, and treatment, apply only to the facial paralysis which is produced by pressure of forceps during delivery.

*Gazette Médicale de Paris.* 1839.

---

*New Method for the radical Cure of Varix, and especially of Varicocele.*
By M. Ricord.

After pointing out the errors of believing that varicocele affects only persons of twenty or thirty years old, and imagining it to be a common consequence of gonorrhœa or epididymitis, whereas in fact it is more generally a predisposing cause of the latter disease, and instead of being produced by it is more often cured by it; M. Ricord proceeds to describe his mode of operation.

"The hair must be shaved from the genital organs on the side to be operated on, and the veins must be dilated by making the patient walk about a little, or by enveloping the scrotum for a few hours in hot poultices, or by fomentations. This being done (though if the swelling is at all times considerable these precautions are unnecessary), the vas deferens must be separated from the mass of veins, and the latter being taken up with a fold of the scrotum, a flat lance-shaped needle armed with a double-looped thread must be passed beneath them. When the needle has been passed completely through the skin from one side to the other, the veins are to be let go, the skin alone being now held up, and then a second needle similarly armed must be passed through over the veins, entering at the same hole by which the first needle was thrust out, and passing out at the same hole by which the first entered. The bundle of veins is thus fixed between two double threads, of which one passes over and the other beneath it. The ends of each double thread on each side are then to be passed into the loop of the other, and now by drawing these ends in opposite directions the vessels are tied beneath the skin. By this kind of ligatures the vessels may either be suddenly constricted or be tied gradually in a manner something like that adopted by M. Breschet, or most conveniently by a properly adopted *serre-nœud* after the fashion of a tourniquet.

"It is usually from the tenth to the twentieth day that the vessels are divided by this means, and their division may be easily recognized by the freedom with which the ligatures may be drawn from one side to the other without being, as they were before, retained by the parts which they inclosed. It sometimes happens that at the instant of the first constriction the patient suddenly feels rather an acute pain in the course of the spermatic cord; it is usually less severe than in the other operations for the same purpose; and though it often recurs at the successive constrictions, yet it has never been long continued, nor given rise to any accident. It is sufficient to keep the scrotum raised, to employ some anodyne frictions on the inguinal canal or the lumbar region, or to apply some emollient poultices, to effect its removal. Sometimes a slight œdema of the scrotum supervenes, and I have twice observed rather a considerable serous effusion in the tunica vaginalis. In one patient also, who went out of the hospital and a few days after the operation exposed himself to great fatigue, a slight abscess formed in the cellular tissue; but with these exceptions there has been no important accident.

"It must be clearly understood that if the patient is strong and plethoric he is to be bled from the arm directly after the operation; that the horizontal position must be maintained till the vessels are cut through; and that the bowels must be carefully kept open.

"Twelve patients have now been operated on in this manner at the Venereal Hospital, and in all the most complete and satisfactory result has been obtained. The three last of them were presented at the Academy of Medicine; two completely cured, and the third, who was operated on only two days previously, still wearing the ligature and the *serre-nœud*.

"I have employed the same method for varices of the legs. I have already operated on nine patients, some having simple varicose swellings, and others varicose ulcers. In some a single ligature was sufficient, in others as many as four were applied. In none have there occurred any symptoms of phlebitis; the varicose veins have been obliterated and the ulcers speedily cicatrized; and in one of the patients whom I saw six months after the operation, there was no relapse. Still, however, I do not think that this method is likely to be so successful in all cases of varices of the lower extremities as in those of varicocele."

*Bulletin Générale de Thérapeutique. Mars*, 1840.

---

*New Plan for Amputating through the Tarsus.* By M. C. SEDILLOT.

THE author proposes by this plan to remedy the inconveniences which have often followed the operation of partial amputation of the foot, as practised by Chopart and modified by Lisfranc and others, in consequence of the wound and the cicatrix succeeding to it having a semicircular form, with its longest diameter opposed to that of the os calcis and astragalus, and commonly extending round three fourths or at least two thirds of the circumference of the stump. This form of wound and cicatrix is constantly produced, whether the operator make both a dorsal and a plantar flap or only the latter. M. Sedillot's plan will undoubtedly remedy this defect, though more experience than has yet been obtained of its success on the living subject is necessary before it can be expected to supersede those in more general use. He describes it briefly as follows:

"The patient lying down or being seated with the leg flexed on the thigh, I find the articulation by the position of the malleoli and the prominences of the os scaphoides and the posterior extremity of the fifth metatarsal bone, of which the distances from the line of articulation are well enough known. Then grasping the dorsal aspect of the foot with my left hand, at the level of the anterior extremities of the metatarsal bones, I place the heel on the edge of a table, so as to have a convenient and firm point of support to stretch the ligaments and separate the articular surfaces as soon as their ligamentous connexions are divided.

"With a small amputating knife I make a first transverse incision, beginning a few lines in front of the calcaneo-cuboid articulation, and terminating on the middle of the dorsal aspect of the foot on the outer side of the tendon of the tibialis anticus. From this latter point I carry a second oblique incision, from behind forwards and from without inwards, which turns round the inner edge of the foot at two fingers' breadth behind the metatarso-phalangeal articulation of the great toe, and is then continued from before backwards, from within outwards, and from above downwards across the plantar aspect of the foot, to the point at which the first incision was commenced. I take care always to divide the integuments of the sole with an oblique bevelled edge, so as to free them as much as possible from the cellular and adipose tissue which might retard their union. I now dissect back the inner flap, which is thus cut out to as far as the tubercle of the os scaphoides, which I take as a guide to open the medio-tarsal articulation, cut the interosseous ligament, slide the knife between the osseous surfaces, and complete the operation by dividing the deep muscles at the level of the incision in the sole."

In this operation the wound, of which the direction corresponds to the longest diameter of the articulation, extends only from the outer edge of the foot to the middle of its dorsal aspect, and though it occupies less than a third of the whole circumference of the foot, yet it is equal to the depth of the calcaneo-cuboid and astragalo-scaphoid articulations; for one of its extremities touches the anterior, inferior, and external edge of the os calcis, and the other the summit of the head of the astragalus. It is therefore on this incision that the cicatrix has afterwards to form; and the skin of the external and superior portion of the foot is alone opposed to the great internal flap, comprising its inferior, internal, and a part of its dorsal aspects. The cicatrix will therefore of necessity be linear and very small.

Another advantage is the smaller quantity of integuments which the medio-tarsal amputation requires. In fact about an inch of skin preserved on the inner side of the foot in front of the os scaphoides is sufficient to cover the bones, and thus the preservation of the foot is rendered possible in cases in which it would otherwise be necessary to amputate the leg. The facility of dividing the tendons of the tibialis anticus, and of the extensors of the first and second toes more anteriorly, is a favorable circumstance for the preventing the drawing-backwards of the stump; and the wound having the same direction as the articular surfaces is well snpported and the more disposed to remain united because the inner flap is formed of integuments which, though not very thick, are sufficiently vascular.

*Gazette Médicale. April* 18, 1840

